# Does the magnetization transfer effect bias chemical exchange saturation transfer effects? Quantifying chemical exchange saturation transfer in the presence of magnetization transfer

**DOI:** 10.1002/mrm.28212

**Published:** 2020-02-18

**Authors:** Alex K. Smith, Kevin J. Ray, James R. Larkin, Martin Craig, Seth A. Smith, Michael A. Chappell

**Affiliations:** ^1^ Wellcome Centre for Integrative Neuroimaging University of Oxford Oxford United Kingdom; ^2^ Cancer Research UK and Medical Research Council Oxford Institute for Radiation Oncology Department of Oncology University of Oxford Oxford United Kingdom; ^3^ Institute of Biomedical Engineering Department of Engineering Science University of Oxford Oxford United Kingdom; ^4^ Vanderbilt University Institute of Imaging Science Vanderbilt University Nashville Tennessee USA; ^5^ Department of Radiology and Radiological Sciences Vanderbilt University Medical Center Nashville Tennessee USA

**Keywords:** amide, CEST, MT, NOE, qMT, quantitative

## Abstract

**Purpose:**

Chemical exchange saturation transfer (CEST) is an MRI technique sensitive to the presence of low‐concentration solute protons exchanging with water. However, magnetization transfer (MT) effects also arise when large semisolid molecules interact with water, which biases CEST parameter estimates if quantitative models do not account for macromolecular effects. This study establishes under what conditions this bias is significant and demonstrates how using an appropriate model provides more accurate quantitative CEST measurements.

**Methods:**

CEST and MT data were acquired in phantoms containing bovine serum albumin and agarose. Several quantitative CEST and MT models were used with the phantom data to demonstrate how underfitting can influence estimates of the CEST effect. CEST and MT data were acquired in healthy volunteers, and a two‐pool model was fit in vivo and in vitro, whereas removing increasing amounts of CEST data to show biases in the CEST analysis also corrupts MT parameter estimates.

**Results:**

When all significant CEST/MT effects were included, the derived parameter estimates for each CEST/MT pool significantly correlated (*P* < .05) with bovine serum albumin/agarose concentration; minimal or negative correlations were found with underfitted data. Additionally, a bootstrap analysis demonstrated that significant biases occur in MT parameter estimates (*P* < .001) when unmodeled CEST data are included in the analysis.

**Conclusions:**

These results indicate that current practices of simultaneously fitting both CEST and MT effects in model‐based analyses can lead to significant bias in all parameter estimates unless a sufficiently detailed model is utilized. Therefore, care must be taken when quantifying CEST and MT effects in vivo by properly modeling data to minimize these biases.

## 1 INTRODUCTION

Chemical exchange saturation transfer (CEST) is a MRI method based on the exchange of magnetization between solutes and water. The protons associated with small, mobile solutes resonate at specific frequency offsets to water, but are difficult to detect directly using MRI, although some are in constant, direct chemical exchange with water.^1^ CEST contrast is generated by selectively saturating the labile protons of mobile solutes using narrow‐bandwidth radiofrequency (RF) irradiation. This saturation is then exchanged with water protons through direct chemical exchange of protons, resulting in an attenuation of the water signal, allowing indirect evaluation of the biochemical constituents of the tissue of interest.^2^, ^3^ Importantly, the magnitude of the attenuation of the water signal is related to both the chemical exchange rate, which has been correlated with pH,^4^ and the concentration of the exchanging solute. CEST imaging is thus a unique imaging technique which can be used to investigate how pathology perturbs the underlying biochemistry and pH balance in the body and has been used to study pathologies such as stroke,^5^, ^6^, ^7^, ^8^ cancer,^9^, ^10^, ^11^, ^12^ and multiple sclerosis.^13^, ^14^

A major confound in CEST imaging is the magnetization transfer (MT) effect. This effect arises from protons attached to immobile, semisolid macromolecules, such as myelin^15^, ^16^ in the central nervous system. The MT effect is characterized as a broad, asymmetric saturation lineshape which introduces significant saturation effects over the entire CEST spectrum.^4^, ^17^ This effect can bias and overwhelm calculations of the CEST effect, such as the MT ratio asymmetry (MTR_asym_) measurement that is routinely performed in CEST analyses.^2^ Increasingly, more detailed models are being implemented to quantify CEST effects and separate different contributions to the spectrum. However, the influence of the MT effect on these analyses has not been systematically explored. While several studies have investigated the MT effect in CEST imaging,^3^, ^18^, ^19^ most often the MT effect is removed as a confound,^20^, ^21^, ^22^ despite it having radiological significance in its own right.^11^, ^23^, ^24^, ^25^, ^26^, ^27^, ^28^, ^29^ Therefore, eliminating this contrast mechanism in favour of CEST may also eliminate valuable information about the tissue under investigation.

Quantitative MT (qMT) imaging has been used to investigate pathologies with underlying macromolecular change by modeling the MT effect using a super‐Lorentzian lineshape.^15^, ^16^ If both labile and macromolecular constituents are affected, collecting both MT and CEST data may provide greater insight into the underlying pathological processes than either method would alone. Previous studies have either quantified the MT effect using a qMT model, and then applied the qMT parameter estimates into a CEST analysis,^11^, ^30^ or simply included the MT effect as an additional pool in a multi‐pool Lorentzian‐lineshape analysis,^31^ preventing comparison of these results with existing qMT literature. To the authors’ knowledge, only one quantitative analysis method, derived from the Bloch‐McConnell equations, has been developed that quantitatively estimates both the MT and CEST parameters simultaneously.^32^, ^33^, ^34^, ^35^ Unfortunately, this methodology has not yet been used to explicitly measure the MT effect, instead modeling it as a combined term with nuclear Overhauser effect‐relayed exchange (NOE).^34^ Simplifying assumptions such as these (eg, combining multiple pools together, ignoring constituents that produce significant CEST/MT effects) may introduce unexpected biases into the analysis, which will lead to inaccurate estimates of CEST parameters.

In the current study, we demonstrate that the biases described above are largely removed when all detectable CEST effects are sufficiently modeled. We illustrate this by (1) expanding Chappell et al’s method^32^ to incorporate a lineshape function, (2) demonstrating this bias in simulations of a seven‐pool model of CEST and MT, (3) fully fitting the model to in vitro data by including all observable CEST pools, and (4) showing that these biases will also influence the parameter estimates of the MT effect using in vitro and in vivo data in a CEST+MT analysis by fitting a two‐pool qMT model with varying amounts of CEST information added to the estimation.

## 2 THEORY

Similar to the analytical solution for a two‐pool model described in the various papers by Yarnykh et al^36^, ^37^ and Zhou et al,^4^, ^38^ we can derive an analytical solution of a multi‐pool CEST model. Without losing generality, we can assume a three‐pool model (free, solute, and semisolid):

(1) $$\frac{{\text{dM}}_{x}^{\text{F,S}}}{\text{dt}}={-(R}_{2}^{\text{F,S}}+k_{\text{FS, SF}})M_{x}^{\text{F,S}}+{2\pi \Delta M}_{y}^{\text{F,S}}+{\;k}_{\text{SF,FS}}M_{x}^{\text{S,F}}$$

(2) $$\frac{{\text{dM}}_{y}^{\text{F,S}}}{\text{dt}}=-\left(R_{2}^{\text{F,S}}{\;+\;k}_{\text{FS, SF}}\right)M_{y}^{\text{F,S}}-2\pi \Delta M_{x}^{\text{F,S}}-\gamma B_{1}\left(t\right)M_{z}^{\text{F,S}}{\;+\;k}_{\text{SF,FS}}M_{y}^{\text{S,F}}$$

(3) $$\frac{{\text{dM}}_{z}^{F}}{\text{dt}}=\gamma B_{1}\left(t\right)M_{y}^{F}-\left(R_{1}^{F}+k_{\text{FS}}+{\;k}_{\text{FM}}\right)M_{z}^{F}{\;+\;k}_{\text{SF}}M_{z}^{S}{\;+\;k}_{\text{MF}}M_{z}^{M}{\;+\;R}_{1}^{F}$$

(4) $$\frac{{\text{dM}}_{z}^{S}}{\text{dt}}={\gamma B}_{1}\left(t\right)M_{y}^{S}-\left(R_{1}^{S}{+\;k}_{\text{SF}}\right)M_{z}^{S}+k_{\text{FS}}M_{z}^{F}+{\;R}_{1}^{S}M_{0,r}^{S}$$

(5) $$\frac{{\text{dM}}_{z}^{M}}{\text{dt}}=-\left(R_{1}^{M}+{\pi \gamma }^{2}B_{1}^{2}\left(t\right)g\left({\Delta ,T}_{2}^{M}\right)+k_{\text{MF}}\right)M_{z}^{M}+k_{\text{FM}}M_{z}^{F}+R_{1}^{M}M_{0,r}^{M}$$

where $M_{\text{x,y,z}}^{\text{F,S,M}}$ are the x‐, y‐, and z‐components of magnetization for the free (F), solute (S), and semisolid (M) pools; ∆ is the frequency offset of the RF pulse with respect to the pool of interest (eg, ${\Delta =\Delta \omega -\delta }_{s}$, where Δω is the RF pulse offset with respect to water, and $\delta_{s}$ is the pool offset with respect to water); B_1_(t) is the amplitude of the RF pulse; $g\left({\Delta ,T}_{2}^{M}\right)$ is the absorption lineshape for the semisolid pool, $R_{1,2}^{\text{F,S,M}}=\frac{1}{T_{1,2}^{\text{F,S,M}}}$; and $k_{\text{AB}}$ is the effective exchange rate from pool A to pool B. Equations (1), (2), (3), (4), (5) are normalized to the equilibrium magnetization of water, $M_{0}^{F}$, and therefore $k_{\text{AB}}{\;=\;k}_{\text{BA}}M_{\text{0,r}}^{B}$, with $M_{0,r}^{B}=\frac{M_{0}^{B}}{M_{0}^{A}}$. The lineshape of the semisolid pool can be represented by several different shapes, including a Lorentzian, super‐Lorentzian, or Gaussian function.^15^, ^16^, ^39^

Recent analyses by Tee et al^33^ demonstrated that a continuous wave equivalent pulse (CWEP) approximation can be used in place of a discretized pulse model with minimal error. This same approximation is used here to simplify the model derivation, and therefore, we can split Equations (1), (2), (3), (4), (5) into the following time intervals: CEST saturation pulse (t_m_), spoiling gradient free precession evolution (t_s_), on‐resonance excitation pulse (t_p_), and the readout and relaxation free precession evolution (t_r_).^36^, ^37^

We can expand the two‐pool model derived by Yarnykh et al^36^, ^37^ to incorporate multiple pools by reintroducing the transverse magnetisation components and eliminating the water saturation function. Using a CWEP approximation, the magnetization vector for a pulsed CEST sequence is similar to that derived by Yarnykh et al,^36^, ^37^ with a few notable differences:

(6) $$M\;={\left({I\;-\;E}_{s}E_{m}{\text{SE}}_{r}\text{CS}\right)}^{-1}\left(E_{s}E_{m}S\left({I\;-\;E}_{r}\right)+{I-E}_{s}+E_{s}\left({I-E}_{m}\right){\left(R_{L}+W\right)}^{-1}R_{L}\right)M_{0}$$

where M is the magnetisation vector of all pools immediately before the excitation pulse, M_0_ is the equilibrium magnetization, defined as $M_{0}=\left[{0,\;0,\;1,\;0,\;0,\;M}_{0,\;r}^{S},\;M_{0,\;r}^{M}\right]$, I is the identity matrix, $E_{m}=\exp\left(\left(R_{L}+W\right)t_{m}\right)$ describes a saturation pulse with duration t_m_, $E_{r}=\exp\left(R_{L}t_{r}\right)$ and $E_{s}=\;\exp\left(R_{L}t_{s}\right)$ describe the free precession intervals following the excitation and CEST saturation, respectively. **C** is a diagonal matrix of the form:

(7) $$C\;=\text{diag}\left(\sin\left(\alpha \right),\text{sin}\left(\alpha \right),\text{cos}\left(\alpha \right),\text{sin}\left(\alpha \right),\text{sin}\left(\alpha \right),\text{cos}\left(\alpha \right),\text{cos}\left(\alpha \right)\right)$$

which corresponds to the instantaneous rotation of the magnetization by an on‐resonance excitation pulse with flip angle α. S is a matrix of the form $S=\text{diag}\left(0,\;0,\;1,\;0,\;0,\;1,\;1\right)$, and is used to spoil the transverse magnetization immediately after each free precession period. The relaxation matrix R_L_ and saturation matrix W are defined as:

(8) $$R_{L}=\left[\begin{array}{ccccccc} {-\;R}_{2}^{F}-k_{\text{FS}} & 0 & 0 & k_{\text{SF}} & 0 & 0 & 0\\ 0 & {-\;R}_{2}^{F}-k_{\text{FS}} & 0 & 0 & k_{\text{SF}} & 0 & 0\\ 0 & 0 & {-R}_{1}^{F}-k_{\text{FS}}{-k}_{\text{FM}} & 0 & 0 & k_{\text{SF}} & k_{\text{MF}}\\ k_{\text{FS}} & 0 & 0 & {-R}_{2}^{S}{-k}_{\text{SF}} & 0 & 0 & 0\\ 0 & k_{\text{FS}} & 0 & 0 & {-R}_{2}^{S}{-k}_{\text{SF}} & 0 & 0\\ 0 & 0 & k_{\text{FS}} & 0 & 0 & {-R}_{1}^{S}{-k}_{\text{SF}} & 0\\ 0 & 0 & k_{\text{FM}} & 0 & 0 & 0 & {-R}_{1}^{M}{-k}_{\text{MF}} \end{array}\right]$$

(9) $$W=\left[\begin{array}{ccccccc} 0 & {2\pi \Delta }_{F} & 0 & 0 & 0 & 0 & 0\\ {-2\pi \Delta }_{F} & 0 & {\gamma B}_{1}\left(t\right) & 0 & 0 & 0 & 0\\ 0 & {-\gamma B}_{1}\left(t\right) & 0 & 0 & 0 & 0 & 0\\ 0 & 0 & 0 & 0 & {2\pi \Delta }_{S} & 0 & 0\\ 0 & 0 & 0 & {-2\pi \Delta }_{s} & 0 & {\gamma B}_{1}\left(t\right) & 0\\ 0 & 0 & 0 & 0 & {-\gamma B}_{1}\left(t\right) & 0 & 0\\ 0 & 0 & 0 & 0 & 0 & 0 & {\pi \gamma }^{2}B_{1}^{2}\left(t\right)g\left({\Delta ,T}_{2}^{M}\right) \end{array}\right]$$

Importantly, the term ${\left(R_{L}+\;W\right)}^{-1}R_{L}$ generalises the M_SS_ term from Equation 12 in Yarnykh,^36^ allowing Equation 6 to be easily expanded to an N‐pool model.

## 3 METHODS

### 3.1 Simulations

All data analyses were performed in Python 3.6.8 (NumPy 1.15.4, Pandas 0.24.1, SciPy 1.2.1, NiBabel 2.3.1). Simulations were performed to demonstrate the bias in the amide and MT estimates resulting from fitting a three‐pool model (free, amide, MT) to a more detailed CEST model (Supporting Information Table S1). A seven‐pool model consisting of water, amide, creatine, three pools representing NOE‐relayed exchange, and a semisolid pool (see Supporting Information Table S1, all pool parameters taken from van Zijl et al^20^) was simulated using the following sequence parameters: a CEST pulse train consisting of fifty 20‐ms Gaussian pulses with a 50% duty cycle, followed by an excitation pulse of 7˚. 36 offsets were simulated between ±5 ppm using a saturation flip angle of 180˚ to acquire the CEST spectrum, and at 7.5, 15, 30, 60, and 100 ppm using saturation flip angles of 180˚ and 540˚ to acquire the MT spectrum. The simulated MT and CEST data were then fit using the variational Bayesian inference algorithm (the Fabber model in FSL v5.0.2.1)^32^, ^40^, ^41^, ^42^ (see Image Processing section) to a three‐pool model (free, amide, semisolid) to demonstrate how the derived CEST and MT parameter estimates are affected by underfitting.

### 3.2 Phantom preparation

**Figure 1:**
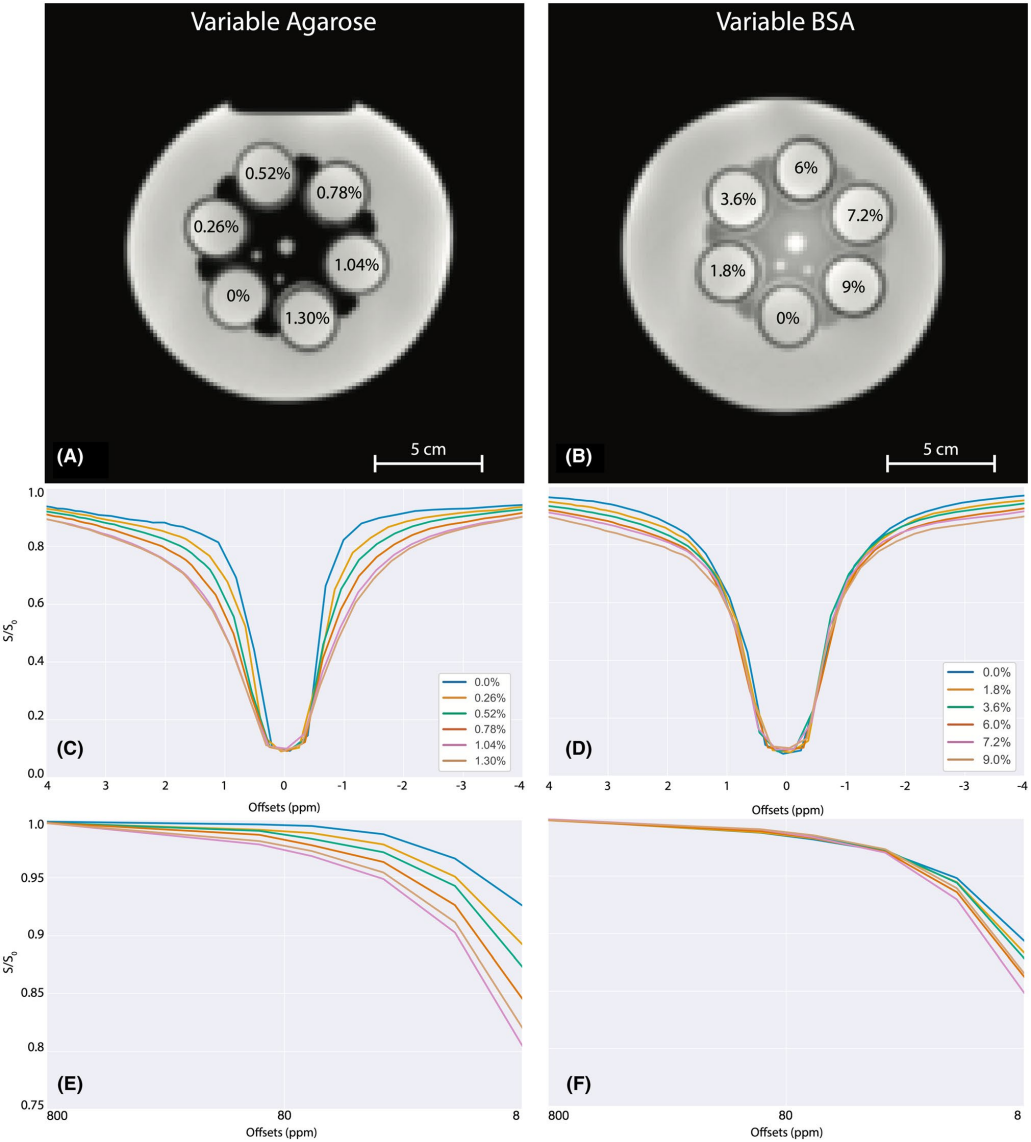
Orientation of the variable agarose phantoms (A) and variable BSA phantoms (B). The percentages in each phantom refer to the w/v of either agarose (A) or BSA (B). The normalized, B_0_‐corrected CEST (C,D) and 540° qMT (E,F) Z‐spectra for the variable agarose (C,E), and variable BSA (D,F) phantoms. CEST Z‐spectra were reduced to ±4 ppm to account for B_0_ shifts in spectra

Two sets of six 50 mL tubes were filled with solutions of phosphate‐buffered saline (PBS) at a pH of 7.4, containing 0.01% w/v NaN_3_ for bacterial suppression, with varying concentrations of agarose gel (A9539; Sigma) and bovine serum albumin (BSA) (A3059; Sigma). The first set of phantoms contained varying concentrations of agarose with 6% w/v BSA. The agarose concentrations were 0%, 0.26%, 0.52%, 0.78%, 1.04%, and 1.3% w/v. The second set of phantoms contained varying concentrations of BSA, with 0.65% w/v agarose. The BSA concentrations were 0%, 1.8%, 3.6%, 7.2%, and 9% w/v BSA, with another copy of the 0.78% w/v agarose, 6% w/v BSA phantom for comparison. Each set of tubes were then placed in custom 3D‐printed phantom holders and set in the centre of two 4L Nalgene bottles (Thermo Fisher Scientific, Waltham, MA, USA). The tubes were then submerged in a bath of deionized water (see Figure 1A,B).

### 3.3 In vivo

Nine healthy volunteers (age: 31.8 ± 7.4 years, 4 female) were recruited with informed consent in accordance with local ethics.

### 3.4 Data acquisition

All images were acquired on a 3T Siemens Verio MRI scanner (Erlangen, Germany) using a single channel transmit body coil and a 32‐channel receive head coil. The same set of images were acquired on both the phantom and in vivo data. The CEST acquisition consisted of a CEST pulse train with a 2D FLASH readout,^43^ with a field of view (FOV) of 220 × 220‐mm^2^, a resolution of 1.7 × 1.7 × 5‐mm^3^, and repetition time/echo time (TR/TE)/ $\alpha_{\text{EX}}$ = 6.1‐ms/2.83‐ms/7˚. The CEST saturation consisted of a train of fifty 20‐ms pulses, flip angle = 184°, 50% duty cycle using 41 frequency offsets asymmetrically sampled between ±4.5 ppm to adequately sample the amide and NOE resonances. Additional offsets at 7.5 ppm, 15 ppm, 30 ppm, 60 ppm, and 100 ppm were acquired to sample the MT spectrum, flip angle = [184˚, 540°]. The shot‐to‐shot interval was 2.375 s. A reference image at an offset of 800 ppm was also acquired at each saturation power. Other parameters included a GRAPPA factor of 3 and 2 averages. A T_1_ map was collected using a variable flip angle 3D‐FLASH sequence (FOV: 220 × 220 × 5‐mm^3^, resolution: 1.7 × 1.7 × 5‐mm^3^, TR/TE/ α = 20‐ms/4.21‐ms/25˚, 20˚, 15˚, 10˚, 5˚). Finally, a $B_{1}^{*}$ map was acquired using the DREAM^44^ sequence (FOV: 256 × 256 × 100‐mm^3^, resolution: 4.0 × 4.0 × 5.0‐mm^3^, TR/TE_1_/TE_2_/ $\alpha_{1}$/ $\alpha_{2}$/ $\alpha_{3}$ = 5‐s/1.29‐ms/2.69‐ms/60˚/8˚/3˚). A high‐resolution T_1_‐weighted image was also acquired to use as a reference (FOV: 220 × 220 × 100‐mm^3^, resolution: 1.1 × 1.1 × 5‐mm^3^, TR/TE/ α= 250 ‐ms/2.48‐ms/70˚). Additionally, to calculate the T_1_ maps for the phantom data, a 2D inversion recovery experiment was performed (FOV: 220 × 220‐mm^2^, resolution: 1.1 × 1.1 × 5‐mm^3^), using inversion times of 100, 300, 500, 1000, 1250, 1750, 2000, 2500, 3000, 3500, and 4000‐ms.

### 3.5 Image processing

#### 3.5.1 Registration

All data were first co‐registered to the CEST image data using tools from FSL v5.0.2.1 (FMRIB, Oxford, UK).^45^ First, the high‐resolution T_1_ image (referred to as HiRes) image was bias‐field corrected using FAST,^46^ and was then brain‐extracted using the brain extraction tool (BET).^47^ The relative slice acquired in the CEST acquisition was identified and extracted from the full HiRes volume, and the HiRes image was then registered to the CEST data using FLIRT with a 2D rigid body registration (the 2D option in FLIRT).^48^, ^49^ Thus, through‐plane motion and/or mismatches in orientation cannot be accounted for when moving from the HiRes reference space to the CEST space. Next, the CEST data were motion‐corrected using MCFLIRT^49^ using the process outlined in Supporting Information Figure S1. The $B_{1}^{*}$ and T_1_ data were registered to the original reference image using FLIRT^48^, ^49^, and the slice that corresponded to the CEST acquisition was extracted from each volume. This slice was then registered using FLIRT^48^, ^49^ to the down‐sampled HiRes volume (and therefore to the CEST image data). The registration pipeline for the full dataset (ie, HiRes downsampling, CEST motion‐correction, and $B_{1}^{*}$ & T_1_ coregistration) can be found at https://github.com/smithalexk/CEST_analysis,hash7f6e655.

#### 3.5.2 Model‐based analysis

Z‐spectra were fit to Equation 7 using the Fabber algorithm in FSL v5.0.2.1 with a customized CEST model (University of Oxford, Oxford, UK) (https://github.com/ibme-qubic/fabber_models_cest,hash4e354a7), which implements a variational Bayesian inference model to perform the estimation.^32^, ^40^, ^41^, ^42^ For each pool, the model fits posteriors based on Bayesian priors of $M_{0,r}^{i}$, $k_{\text{iw}}$, and $T_{2}^{i}$. As the T_1_ for each pool is difficult to characterise independently, the T_1_ prior is set to the T_1,obs_ value determined from the variable flip angle T_1_ data, as has been done previously.^24^, ^50^ The model also accounts for $B_{1}^{+}$ inhomogeneities, by setting the $B_{1}^{+}$ prior to the $B_{1}^{+}$ map derived from the DREAM sequence. Importantly, the implementation of Fabber used here adds the ability to select different MT saturation lineshapes, as well as calculates Z‐spectra for excitation flip angles other than 90˚. In all analyses, the semisolid pool in phantom data was fit with a Gaussian lineshape,^3^ whereas the in vivo semisolid pool was fit with a super‐Lorentzian lineshape.^15^, ^16^ Furthermore, to align the MT effect more closely with qMT literature, the relative pool concentration $M_{0,r}^{\text{MT}}$ is referred to as the semisolid to water pool size ratio (PSR).^51^, ^52^

#### 3.5.3 Multi‐pool phantom analysis

BSA has been shown to have three CEST peaks: an amide resonance centred at 3.5 ppm, a fast‐exchanging resonance centred at 2.0 ppm, and a peak from relayed‐NOE exchange centred on −3.5 ppm.^53^, ^54^ However, as the guanidinium protons exchange at approximately 1000 Hz, it is most likely not detectable at 3T, particularly using when using low‐power pulses,^2^ and was thus excluded from the analysis. We therefore fit a four‐pool model (free, amide, NOE, semisolid) to the CEST+MT data to demonstrate that biases due to the MT effect are largely removed when all detectable CEST effects are included. We also analysed the data by estimating the water and MT pool parameters first using the >5 ppm data similar to Mehrabian et al^11^; these parameters were then fixed during the CEST parameter estimation to demonstrate that the estimates derived from a qMT analysis can also be used to correct CEST parameter estimations in a simplified CEST model. These two approaches to a multi‐pool analysis are referred to as the CEST+MT and qMT‐Fix analyses, respectively. For the CEST data, the MTR*, a measure of the MTR_asym_ in an ideal two‐pool model, was generated for each pool (amide, NOE), as has been described previously,^32^ while the semisolid pool was described using the PSR. In order to generate the MTR*, the exchange rate and concentration for each pool output from the Bayesian analysis were used to generate an idealized two‐pool Z‐spectrum, and the MTR* was calculated using Equation 10.^32^ The calculation compares the signal at the CEST pool frequency from this two pool Z‐spectrum [ $S_{F+S}\left(\Delta \omega \right)$] with the signal from an idealized one‐pool Z‐spectrum [ $S_{w\left(\Delta \omega \right)}$], normalized by the unsaturated signal ( $S_{0}$). The T_1_ and T_2_ relaxation values in the idealized simulations were set to 1.0 s and 140 ms for the water pool, to 1 s and 10 ms for the amide pool, and to 1 s and 5 ms for the NOE pool. The three‐pool fit described by Tee et al^34^ (water, amide, NOE+MT) was used to demonstrate the importance of estimating the MT pool as a distinct pool (referred to as the NOE+MT analysis).

(10) $${\text{MTR}}^{*}=\frac{S_{w}\left(\Delta \omega \right)-S_{F+W}\left(\Delta \omega \right)}{S_{0}}$$

#### 3.5.4 Bootstrap analysis

To demonstrate the bias caused by failing to include detectable CEST effects is not limited to the CEST pool, but can, indeed, affect the MT pool, a two‐pool model (water, semisolid) was repeatedly fit to the in vivo and phantom CEST+MT data, after removing 10 pseudo‐random offsets at a time from the CEST data, in a bootstrapping analysis (five iterations, including 41, 31, 21, 11, and 1 offsets). To correct for B_0_ inhomogeneities, the B_0_ map posterior from the full CEST+MT dataset was used as an image prior for all reduced datasets. This produced five independent posterior estimations (as a function of the number of CEST offsets included) of the two‐pool parameter estimates. These posteriors were then compared to two‐pool posteriors derived from qMT‐only data.

### 3.6 Statistical analysis

Regions of interest (ROIs) were drawn in the splenium, genu, caudate, and thalamus using the HiRes image for each volunteer and in each phantom using MIPAV (NIH, Bethesda, MD, USA), and mean parameter values were taken from each ROI.

For the multi‐pool analysis, the MTR* (amide, NOE) or PSR (MT) was correlated with either the BSA or agarose concentration and the coefficient of determination (*R* ^2^) was calculated (using SciPy’s linregress function). The Wilcoxon rank‐sum test was used to statistically compare the PSR, semisolid‐to‐water exchange rate (k_MF_), and semisolid T_2_ (T_2M_) between the qMT‐only data and the bootstrapped CEST+MT data.

## 4 RESULTS

### 4.1 Simulations

**Figure 2:**
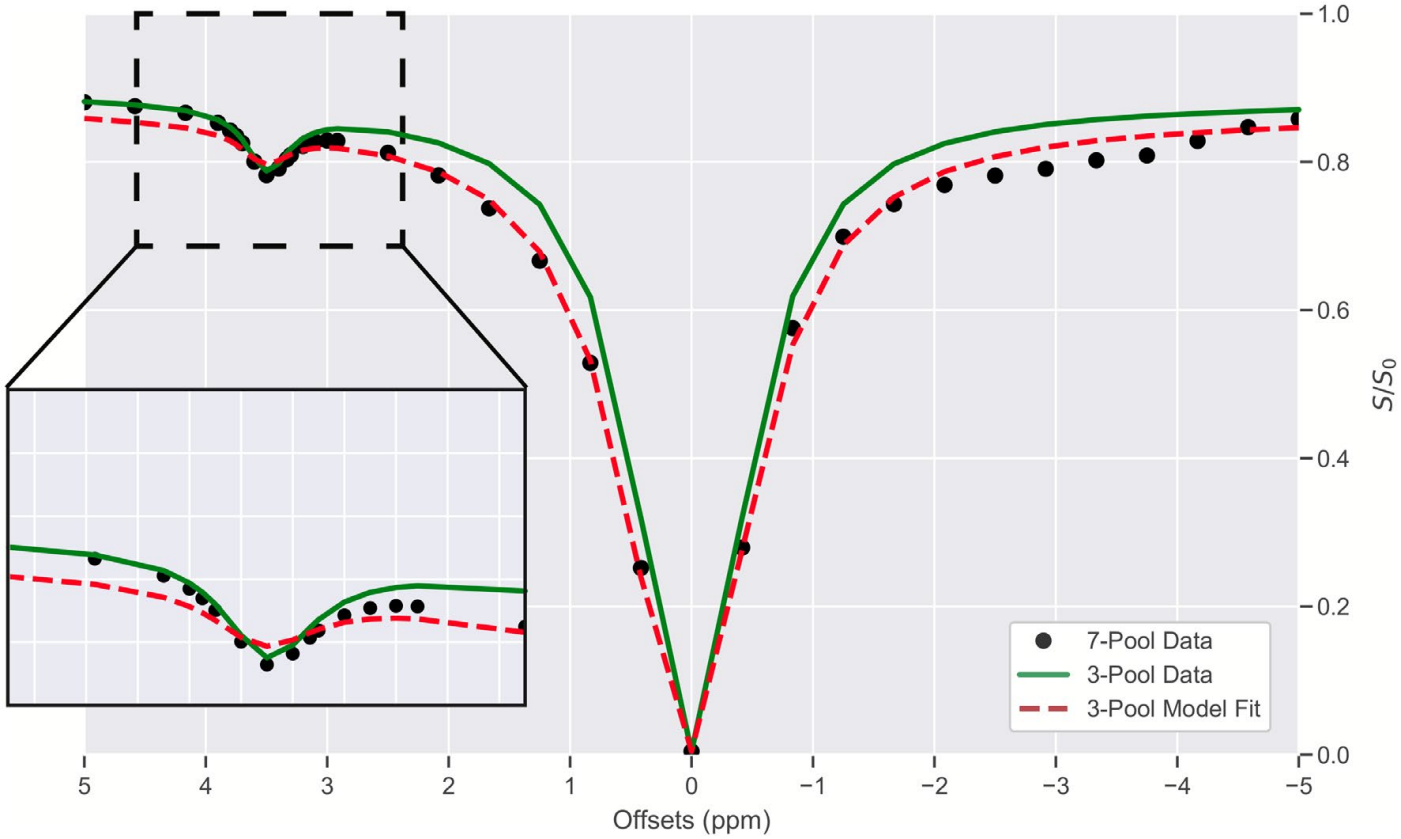
Simulation results demonstrating the bias. The black points represent a Z‐spectrum using seven pools (free, amide, creatine, 3 NOE‐relayed exchange peaks, semisolid), whereas the green line represents a three‐pool Z‐spectrum (free, amide, semisolid). Using a model that does not adequately represent the underlying biochemical environment (eg, using a three‐pool model to fit seven‐pool data, such as that represented by the red dashed line) may result in underfitting, and therefore, will bias results

The Z‐spectra for the simulations are displayed in Figure 2. Comparing the seven‐pool data (black dots) to the three‐pool data (solid green line), the effect of the extra CEST peaks is realized as a spectrum‐wide reduction in relative signal, particularly on the upfield side of the spectrum, where the addition of NOE pools produces a broad decrease in signal. The effect of fitting a three‐pool model to seven‐pool data is also illustrated (dashed red line). While the deviations from the seven‐pool data are minimal, the fit surrounding the amide peak is overestimated (see Figure 2 inset), while the fit on the upfield side of the spectrum is poor throughout. This introduces a bias into the estimated PSR (PSR = 15.26%), relative to the simulated PSR (PSR = 20%). Additionally, while the observed PSR bias is relatively small ( ∼20% bias), more significant changes were observed in the three‐pool fit of MTR*, relative to the simulated MTR* (5.2% and 11.0%, respectively, >50% bias).

### 4.2 Four pool analysis

#### 4.2.1 Phantom data

Figure 1C‐F displays the CEST and qMT Z‐spectra for each set of phantoms. In the variable agarose phantom (Figure 1A,C,E), both the CEST and qMT spectra are affected as the amount of agarose (and therefore semisolid concentration) is increased, reflecting the broad spectral influence of the semisolid component. Conversely, the variable BSA phantom (Figure 1B,D,F) only displays significant variations within the CEST spectrum, reflecting the increasing amide and NOE concentrations.

**Figure 3:**
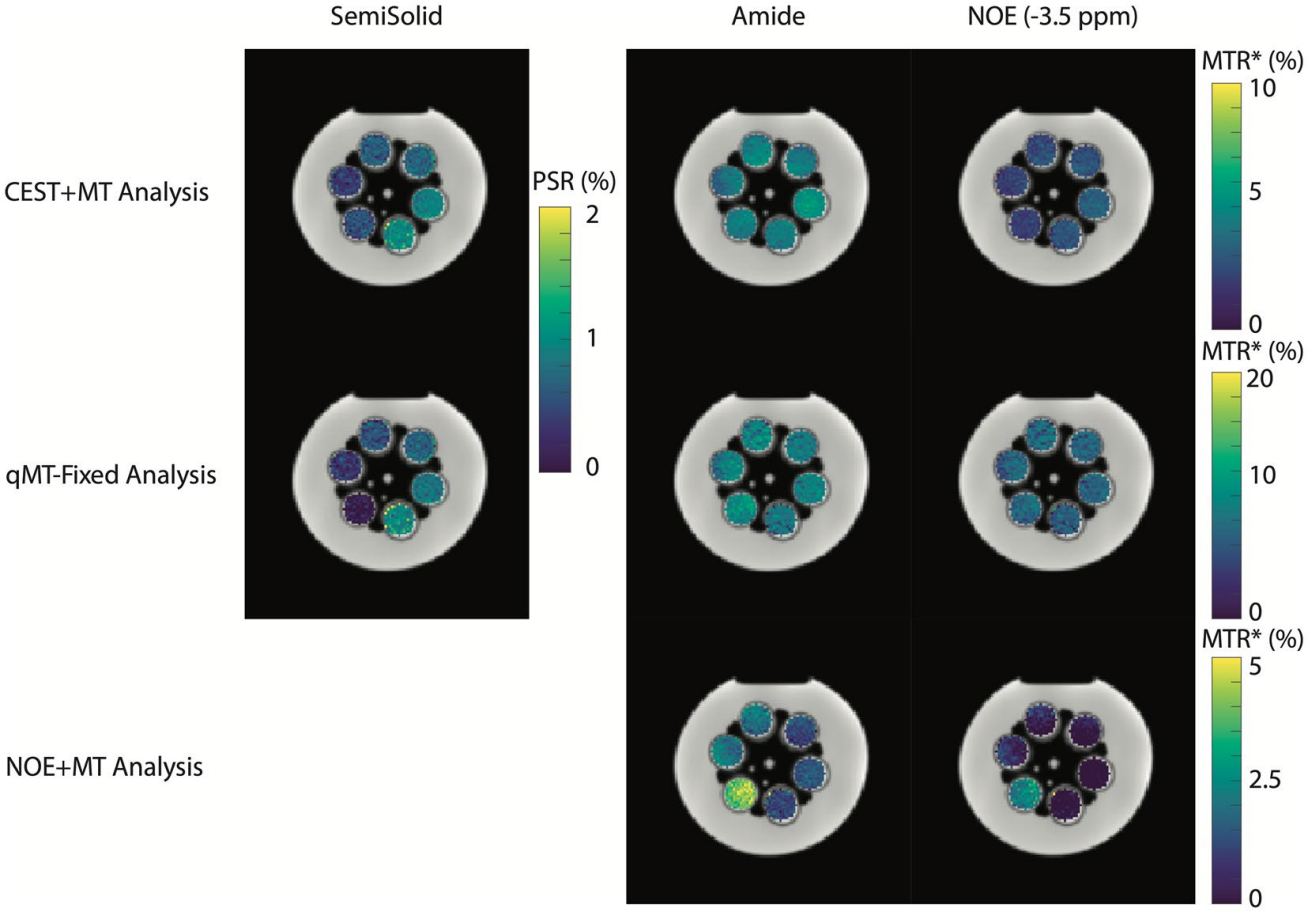
Semisolid (PSR), and amide and NOE (MTR*) maps for the variable agarose phantom experiment. The top row illustrates the full, four‐pool model estimation, the middle displays the fixed‐qMT estimation, and the bottom row shows the same fit assuming the NOE and MT pools are a single pool, similar to Tee et al^34^

**Figure 4:**
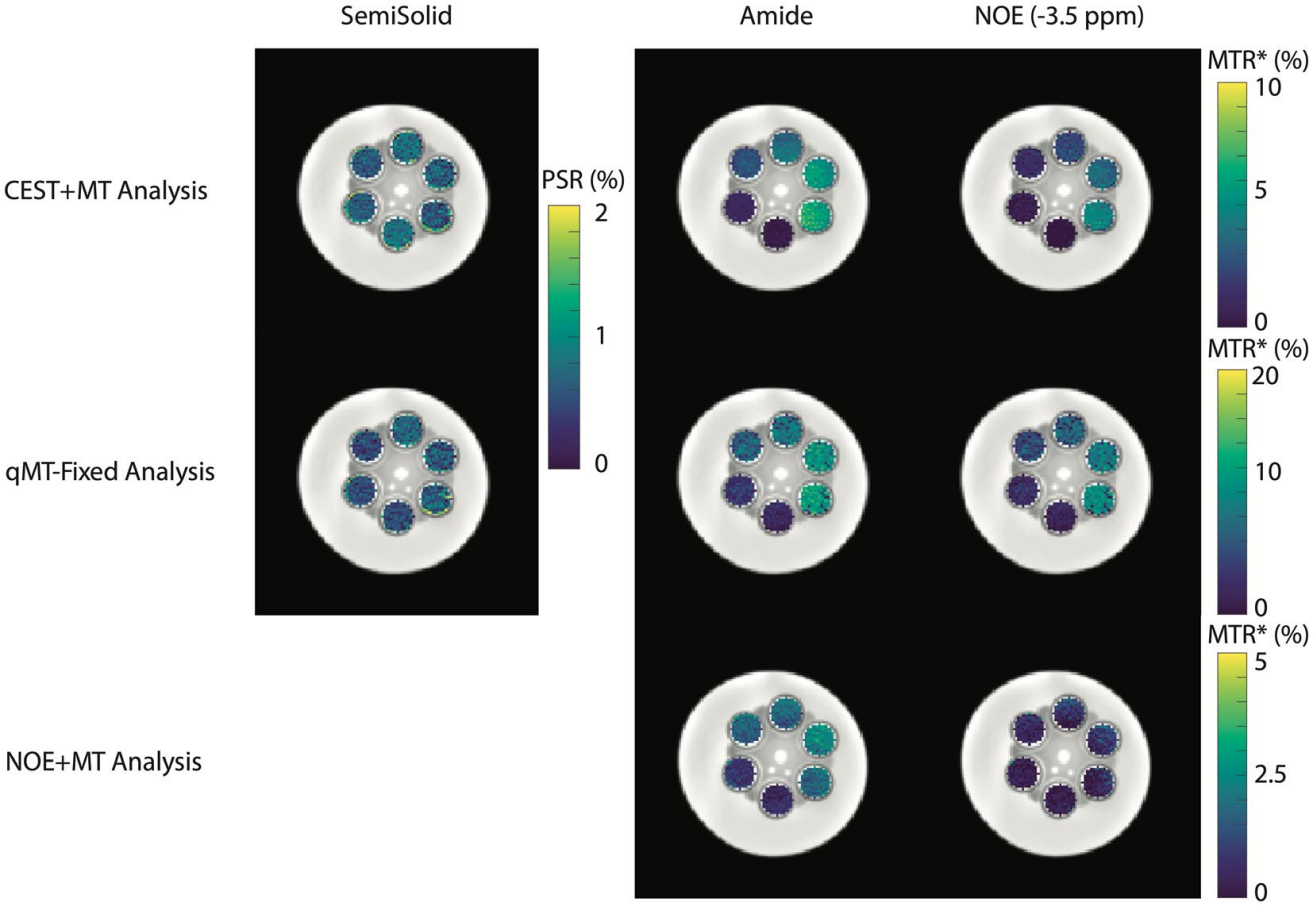
Semisolid (PSR), and amide and NOE (MTR*) maps for the variable BSA phantom experiment. The top row illustrates the full, four‐pool model estimation, the middle displays the fixed‐qMT estimation, and the bottom row shows the same fit assuming the NOE and MT pools are a single pool, similar to similar to Tee et al^34^

Two methods to potentially alleviate the biases introduced by underfitting CEST data are shown in Figures 3 and 4, where a four pool (water, amide, −3.5‐ppm NOE, semisolid) model estimation is performed using the CEST+MT (top row) and the qMT‐Fix (middle row) methods. The bottom row of Figures 3 and 4 illustrates the parameter estimates from the underfit NOE+MT analysis. Figure 3 displays the results of these three approaches for the variable agarose phantom, while Figure 4 shows the results for the variable BSA phantom. Importantly, the PSR estimates for each phantom were similar across each methodology that included a distinct MT pool, indicating that including the amide and NOE pools in the analysis has significantly reduced the biases that were present in the two‐pool model. Additionally, while the amide and NOE MTR* parameter estimates from the CEST+MT analysis were lower than those found using the qMT‐Fixed analysis, the removal of the MT pool caused considerable changes to the estimated MTR*, particularly in the variable agarose phantoms. In particular, the amide MTR* changed dramatically as the agarose concentration increased, indicating that accurate quantification of the MT effect is imperative to ensure the stability of the CEST parameter estimation.

**Figure 5:**
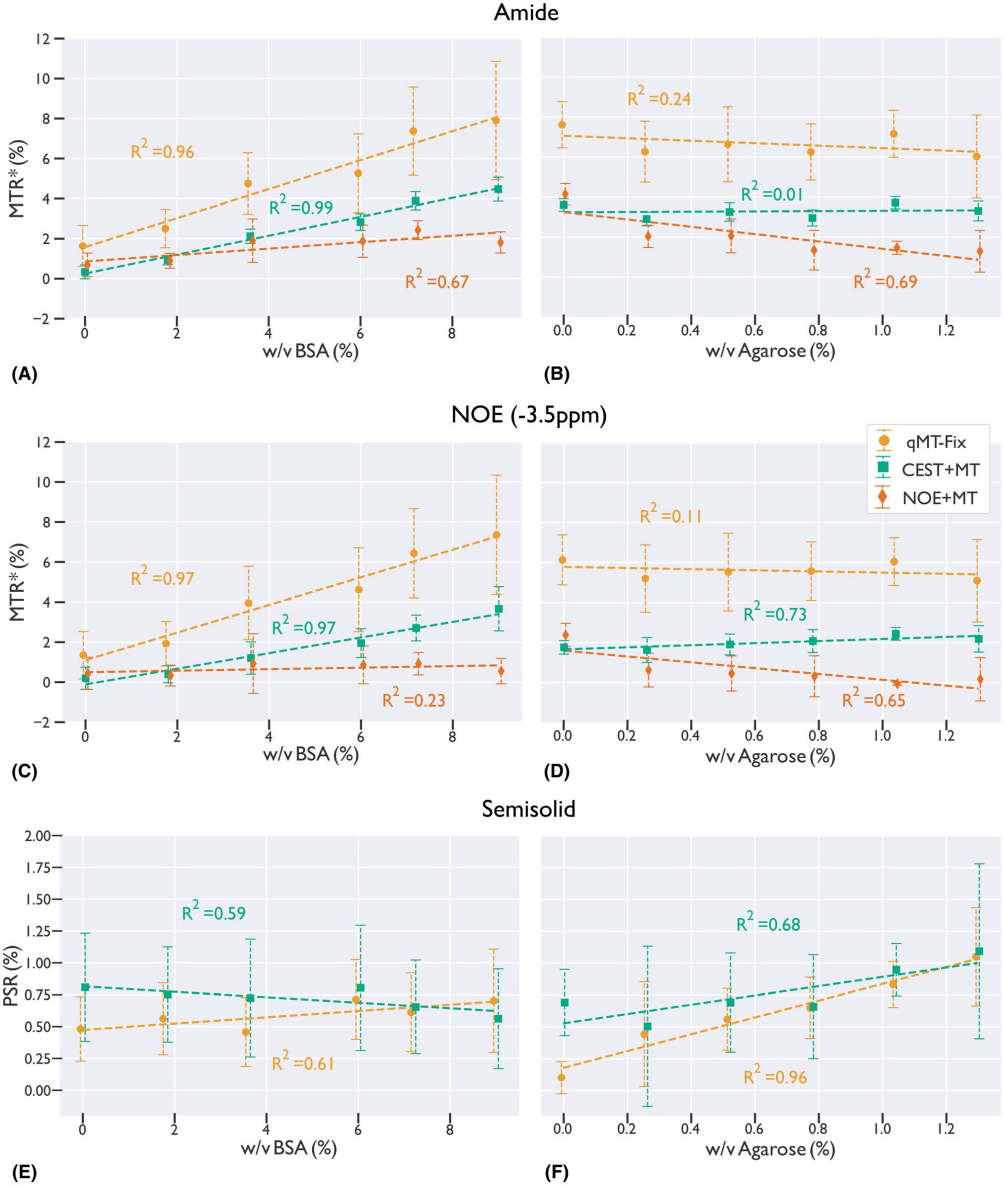
Correlation plots for each full‐model fit as a function of both BSA concentration (A,C,E) and agarose concentration (B,D,F) for the amide (A,B), NOE (C,D), and semisolid (E,F) pools. Dotted lines display the trendlines for the respective correlation. The amide and NOE MTR* are strongly correlated with BSA concentration for the qMT‐Fix and CEST+MT models (*R* ^2 ^> 0.9). The semisolid PSR in F is strongly correlated with agarose when using the qMT‐Fix, but not with the CEST+MT. However, removing the 0.0% agarose point increase the correlation to *R* ^2 ^= 0.92

The differences between these methods can be seen in more detail in Figure 5, which shows the correlation of each moiety with BSA and agarose concentration, and in Table 1, which displays the significance levels for these correlations. Using either the CEST+MT or qMT‐Fix analyses produces strong correlations in the amide and NOE MTR* (Figure 2, 5A,C, *R* ^2^ > 0.9, *P* < .001), with only minimal, non‐significant correlations in the MT pool (Figure 2, 5E, *R* ^2^ ≈ 0.6, *P* > .05). The opposite trend is seen with increasing agarose concentration, where the MT pool shows high correlation (Figure 2, 5F, *R* ^2^ = 0.9, *P* < .05), while the NOE and amide MTR* show low correlations (Figure 5B,D, max *R* ^2 ^= .73, *P* > .05, except for CEST+MT NOE: *P* = .03). Note that, while the semisolid *R* ^2^ for the CEST+MT analysis is nominally 0.68, this is due solely to the high PSR estimate at 0% agarose. Removing this point from the correlation increases *R* ^2^ to 0.92 (*P* = .011), in line with the other correlations.

**Table 1:** *P*‐values associated with the correlation plots from Figure 5

	Variable BSA phantoms			Variable agarose phantoms		
	qMT‐Fix	CEST+MT	NOE+MT	qMT‐Fix	CEST+MT	NOE+MT
Amide	**<0.001**	**<0.001**	**0.045**	0.322	0.841	**0.041**
NOE	**<0.001**	**<0.001**	0.330	0.523	**0.030**	0.054
Semisolid	0.068	0.073	–	**<0.001**	**0.045**	**–**

*Note*: Bolded values represent significant correlations (*P* < .05). Similar to Figure 5F, when removing the 0.0% agarose point the *P*‐value for the correlation is .011.

Conversely, when combining the NOE and MT pools, there is no clear correlation in any pool (Figure 5A‐D, all *R* ^2^ ≤ 0.69 *P* > .04), with some of the pools displaying negative trends as the concentration of either agarose or BSA are increased. This indicates that not fully describing the CEST environment, or incorrectly modeling it, can cause severe disparities, which can affect all moieties under consideration.

### 4.3 Bootstrap analysis

**Figure 6:**
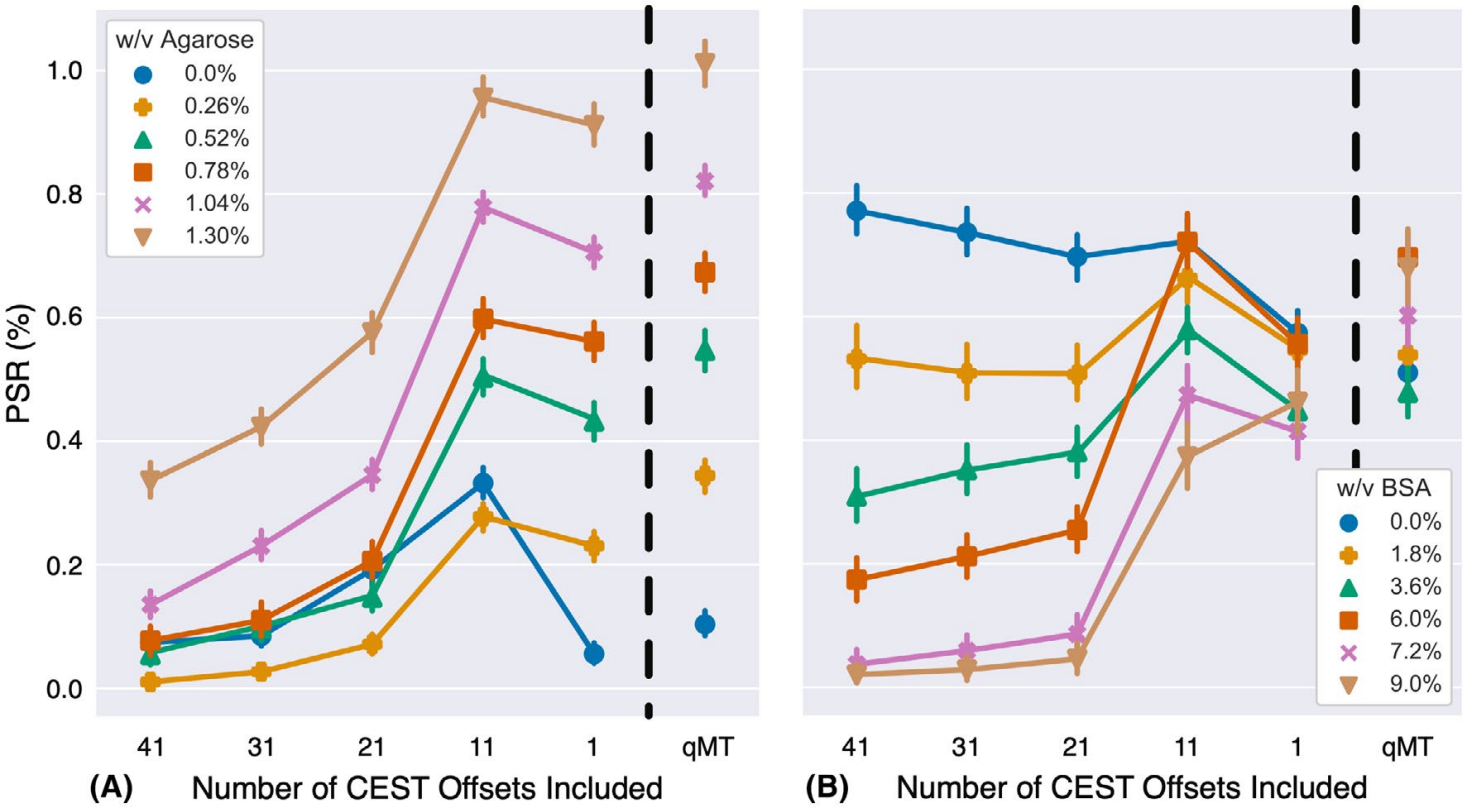
PSR as a function of the number of CEST data‐points used in the analysis for the phantoms with varying agarose (A) and the phantoms with varying BSA (B). The dashed line indicates the split between data with CEST information, and qMT‐only data. As more CEST information is added to the analysis, the PSR derived from the experiment is biased. The error bars represent the standard deviation for each PSR estimate

Figure 6 illustrates the effect of unmodeled CEST data on the estimated PSR. As the number of CEST offsets included in the model decreases, the PSR approaches the values estimated by the qMT‐only points, becoming most similar when a significant amount of CEST data are removed (ie, with less than 10 CEST offsets remaining). Indeed, most of the phantom data PSR is reduced with respect to the qMT‐only measurement, with only the 0% agarose data deviating from this trend. However, this deviation is most likely due to the fitting attempting to create an MT pool when no such pool is present, and therefore, any significant deviation from water‐only is treated as the MT pool (eg, the saturation from amides and NOE). Furthermore, as BSA concentration increases (Figure 6B), the bias in the estimated PSR also increases as more CEST information is introduced to the analysis; for instance, the bias in PSR is lower in the 0% BSA data (blue) relative to the 9% BSA data (brown).

#### 4.3.1 In vivo data

**Figure 7:**
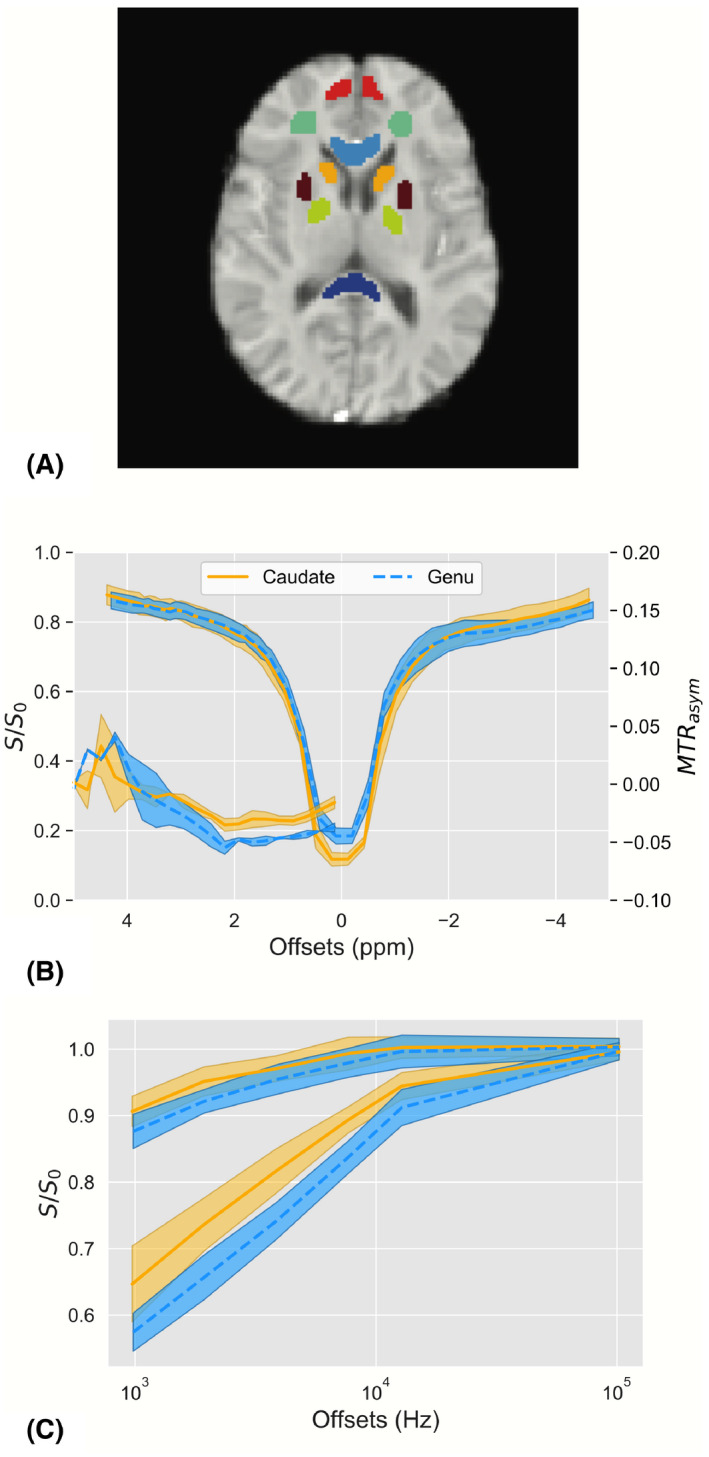
A, Reference image with ROIs of the genu (light blue), splenium (dark blue), caudate (yellow), frontal white matter (green), internal capsule (dark red), frontal gray matter (light red), caudate (yellow), and caudate (gold). Plots of the CEST (B) and MT (C) spectra for the caudate (solid, gold) and genu (dashed, light‐blue). The shaded areas for each plot represent the standard deviation over each point. The MTR_asym_ for each ROI has also been plotted for the CEST data

**Figure 8:**
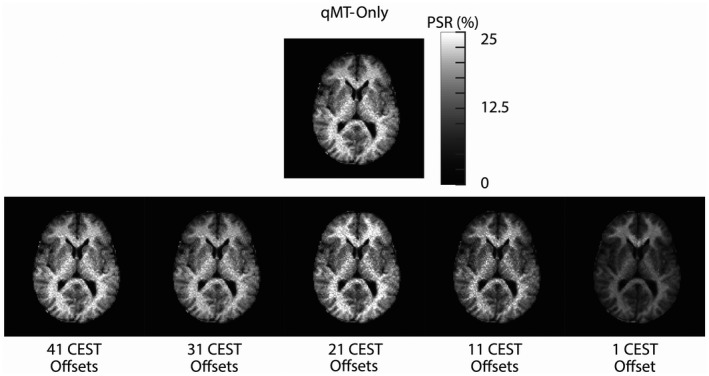
The estimated PSR using a two‐pool model (water, semisolid) for a single volunteer for the qMT‐only data (top), and as a function of the number of CEST offsets (bottom). As more CEST data are included in the qMT analysis, estimates of both gray and white matter PSR significantly increase

An example of the ROIs drawn on the HiRes image, along with example in‐vivo Z‐spectra in the caudate and genu, are shown in Figure 7. An example B1 transmit map is shown in Supporting Information Figure S2. The genu and caudate display similar CEST saturation effects; however, the Z‐spectrum in the genu is more asymmetric about the water resonance (Figure 7B, MTR_asym_), and produces a larger MT effect (Figure 7C), consistent with the genu containing more myelin than the caudate. This observation is confirmed in the qMT‐only PSR estimate shown in Figure 8 (top row), where we see much higher PSR in the genu relative to the caudate. However, this contrast difference becomes less obvious as we add more CEST data point to the parameter estimation (Figure 8, bottom row), where the PSR estimates in both the white matter and gray matter increase as more unmodeled CEST data are added to the analysis. Critically, this effect is observed across the brain, indicating that unmodeled CEST effects are affecting the estimates of the PSR.

**Figure 9:**
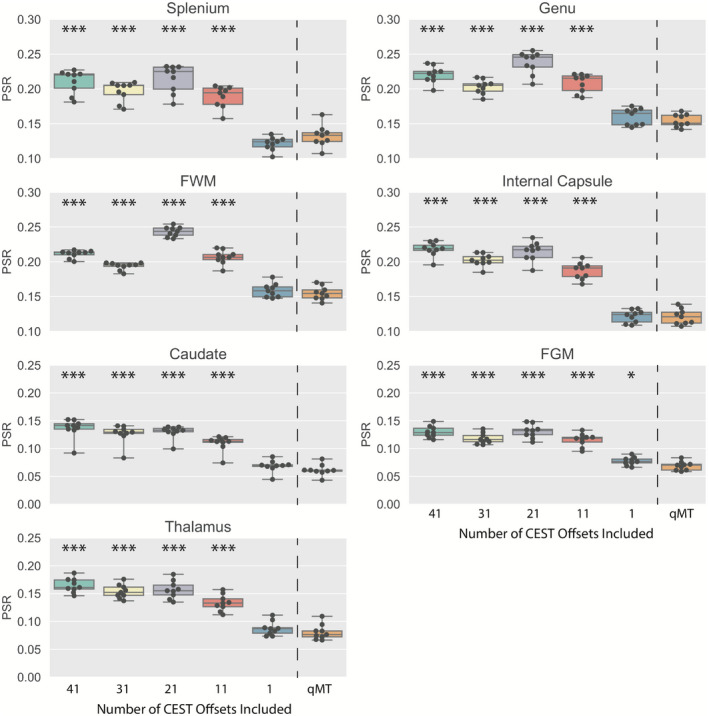
Boxplots displaying the mean PSR as a function of the number of CEST offsets for the splenium, genu, frontal white matter (FWM), internal capsule, caudate, frontal gray matter (FGM), and thalamus, with the qMT‐only fits as a comparison (to the right of the dotted line). Significant differences are also displayed (*P < .05, ***P < 10^−3^)

The bias introduced by unmodeled CEST effects is further demonstrated in Figure 9. The PSR across all ROIs is significantly different from the qMT‐only data if any CEST data are included in the analysis (*P* < 10^−3^ for more than 1 CEST offset). Indeed, the qMT‐only data matches established values for PSR in the brain structures highlighted,^50^, ^55^ indicating that the unmodeled CEST effects are producing bias in the analysis. This is further confirmed in the k_MF_ and T_2M_ estimates (Supporting Information Tables S2‐S3), where each parameter is significantly different (*P* < 10^−3^) once substantial amounts of CEST data are included in the analysis.

## 5 DISCUSSION

The goal of this study was to demonstrate that significant biases can appear in quantitative models of CEST and MT if care is not taken to properly include all significant CEST and MT effects. We demonstrated that utilizing a model that is not sufficiently detailed to estimate the underlying exchange processes will cause biases to occur (Figure 2) in the parameters derived from the CEST and MT pools under consideration. We then confirmed that these biases can be removed if a sufficiently realistic model is applied to the data (Figures 3–5, Table 1), as well as how improper modeling of the MT effect influences the parameter estimations for the observable CEST effects. We finally showed that these effects are not limited to the CEST pools, but that the model parameters for the MT pool will be biased if all observable CEST effects are not included in the model (Figures 6, 8, and 9).

### 5.1 Model‐based analyses

The MT effect is a major confound in all CEST imaging studies^2^, ^20^ if not properly modeled; however, it is also a well‐understood biophysical process. The literature quantifying the MT effect is vast, with many qMT studies in healthy^50^, ^56^, ^57^, ^58^, ^59^ and pathological^24^, ^25^, ^26^, ^27^, ^60^, ^61^ tissues in the human body; thus, it has a well‐defined parameter range. However, most previous studies of in vivo CEST effects have either ignored the MT effect (eg, using the MTR_asym_), or utilized an alternative fitting model, such as AREX,^5^, ^62^ to simultaneously estimate MT and CEST effects. Unfortunately, neither of these methods allows validation of the MT effect with existing qMT literature; there is either no parameter to investigate the MT effect (MTR_asym_) or the parameter describing the MT effect is derived using different methodologies (AREX), meaning it has a different parameter range than what has already been established in the literature. By harmonizing the estimates derived from a CEST analysis with established methods in the qMT literature, we can better correlate the structural (qMT) and chemical (CEST) environments, particularly in pathologies such as multiple sclerosis,^13^, ^14^, ^24^ and cancer.^11^, ^63^

When performing a model‐based fitting of the Bloch‐McConnell equations, care must be taken to ensure biases are not introduced into the parameter estimates. This is particularly true for tissues that have a significant MT effect. The broad spectral linewidth of the MT effect ensures that it will absorb any unmodeled CEST effects (Figures 2, and 8). Unfortunately, most in vivo CEST experiments will be influenced to some degree by the MT effect. This can be seen in Figure 5, where the amide MTR* changes drastically as a function of the agarose concentration. By using a sufficiently detailed model, we can reduce this bias.

This work expands upon previous work by Desmond and Stanisz.^3^ They demonstrated that CEST and MT can be quantified simultaneously using the Bloch‐McConnell while varying pH; however, they limited their analysis to constant CEST and MT solute concentrations, and they only utilized a single CEST pool in their analyses. The results here demonstrate that we can accurately quantify multiple CEST and MT effects while ensuring the effect from each pool is isolated.

This paper presents two methods for overcoming the biases introduced by the MT effect: fully modeling the biophysical environment, or independently modeling the MT effect. While either method provides complementary information to estimate CEST and MT effects, simultaneously modeling all observable pools is preferred over modeling the MT effect separately, particularly when using a Bayesian approach. Under the Bayesian model, it is more accurate to allow all parameters of interest to vary; therefore, fixing the MT pool beforehand could introduce bias into the model. However, it is important to note that the MT effect could be influenced by the more accurate estimation of the water pool T_2_. Therefore, if comparing estimates of the MT effect with previous, traditionally acquired MT data (such as that shown in Sled and Pike^56^), it would be prudent to fix the MT effect first to ensure these comparisons are accurate.

When considering quantifying the CEST effect, care must be taken to avoid overfitting the data. Ideally, the most accurate method would be to fully model the environment under observation; however, this may be difficult to perform for in vivo data. The number of pools necessary to completely model an in vivo metabolic environment would produce a model that is strongly susceptible to overfitting, particularly if pools are included in an experiment where they are not detectable (eg, fast exchanging pools in a low‐power, low‐duty cycle scheme). The effect of overfitting can be seen in Supporting Information Figure S3, where a hydroxyl pool (exchange rate = 2000 Hz) was added to the four‐pool model previously utilized for the CEST+MT, qMT‐Fix, and NOE+MT analyses. The CEST+MT analysis displays strong correlations (*R* ^2^ > 0.85) for every pool as a function of BSA concentration as well as for every pool except amides (*R* ^2^ = 0.55) as a function of agarose concentration (*R* ^2^ > 0.80). Furthermore, the MTR* for the amide, NOE, and hydroxyl pools in the qMT‐Fix and NOE+MT analyses contain almost identical values. This implies that fixing the MT model will produce the same estimations as assuming the MT effect is due to NOE, which is not correct given the moieties under consideration. Therefore, it is crucial to carefully consider which CEST pools are predicted to contribute significant contrast to the experiment in question to avoid overfitting the data. Fitting the MT effect independently may reduce overfitting in the CEST domain and reduce bias in the estimated CEST parameters; indeed, a similar analysis has been performed previously.^11^

### 5.2 Limitations

One limitation of the study is the lack of parity between the qMT‐Fix and CEST+MT techniques in Figures 3 and 4. Estimating the MT pool beforehand may remove some of the interplay between the different parameter values during the Bayesian inference, which may cause these differences to arise. Additionally, because the CEST data were only collected over a single saturation power, it may difficult to accurately separate the exchange rate and M_0_ components of the model.^64^, ^65^ Therefore, utilizing multiple CEST saturation powers over the target solute region may help reduce the disparity between the two methods. However, while the effect magnitudes are different, it is important to note that the relative effect sizes accurately depict the changes in CEST or MT effect for both methods. This is in contrast to the NOE+MT correlation plots in Figure 5a‐d, where the MT effect is combined with the NOE. Here, both the amide and NOE CEST effects are clearly corrupted by the MT effect, demonstrating that small variations in the CEST effect are beneficial to an otherwise completely inaccurate parameter estimation.

Implementing the analysis methods presented here also requires several additional scans in order to quantitatively model the CEST effect. This means either increasing scan time or reducing the amount of CEST data that is acquired for a given resolution and FOV. This may make it difficult to develop a clinically applicable CEST scan. However, several other methods already require these scans.^11^, ^66^ Furthermore, previous studies have been performed that collected CEST data in a clinically acceptable scan time (≈3 min),^7^, ^34^ and new sequences have been developed that may significantly reduce the scan time for a given imaging volume.^66^ Thus, moving to a quantitative analysis methodology may not preclude acquiring CEST data in a clinical setting.

While we do not compare the model‐based analysis with multi‐pool Lorentzian models,^5^, ^67^, ^68^, ^69^ these models are only semi‐quantitative, as the CEST parameter estimates derived from such models are dependent upon the B_1_ amplitude associated with the CEST acquisition. Therefore, changing the sequence parameters may also influence the parameter contrast. This effect should largely be alleviated by quantitatively estimating the data using the Bloch‐McConnell equations, which utilizes the B_1_ amplitude to estimate the CEST effect. Additionally, the MT effect has been shown to be modeled as a super‐Lorentzian in vivo^15^, ^16^; however, this would not be the case in a multi‐pool Lorentzian model, introducing further confounds into a comparison between each methodology. Importantly, Mehrabian et al^11^ demonstrate one method to combine the multi‐pool Lorentzian with qMT, by solving for the qMT parameters and then removing the MT effect from the data, before fitting the remaining CEST data using a multi‐pool Lorentzian approach. This may, indeed be a useful approach, as it allows for direct comparisons with existing MT and CEST studies, while reducing the biases associated with each methodology.

Other researchers have developed complementary techniques to quantify the CEST and MT effects simultaneously. Zaiss et al^19^ demonstrated that their $R_{1\rho }$ method can fit both CEST and MT data; importantly, this is an analytical model and, thus, may converge more rapidly than using the Bloch‐McConnell equations, which require multiple matrix multiplications during the fitting process. However, incorporating more pools may require re‐derivation of the model, which could prove difficult if several CEST effects are expected. Malik et al^70^ recently expanded extended phase graph theory to include an MT pool, and Chan et al^71^ adapted the extended phase graph model to quantify both CEST and MT effects. They found good agreement of their model with their phantom concentrations and were able to apply their methods in vivo. Importantly, the methods presented here can be adapted to use Chan et al’s extended phase graph theory,^71^ which could further improve the model fittings. Comparing these methods, as well as expanding the analysis to incorporate multiple CEST saturation schemes in order to isolate CEST effects with different exchange rates, is the subject of future work.

## 6 CONCLUSIONS

In conclusion, the results of this study demonstrate that quantitative models of the CEST effect in vivo may be biased by the MT effect due to underfitting. While methods do exist to correct this bias, care must be taken when modeling CEST and MT effects in vivo by either fitting MT data separately, then propagating these estimates into the CEST analysis, or by employing a sufficiently detailed model that minimises these biases*.*

## Supporting information


**TABLE S1** Pool Parameters used in simulations. T_1_ values were taken from the average of the T_1_ in the genu of a single subject. All other parameters were taken from van Zijl et al (1)
**TABLE S2** Mean free‐to‐macromolecular exchange rate as a function of the number of CEST offsets for the splenium, genu, frontal white matter (FWM), internal capsule (IC), caudate, frontal grey matter (FGM), and thalamus, with the qMT‐only fits as a comparison. Fits which are significantly different to the qMT‐only data are in bold (*P* < 10^−3^)
**TABLE S3** Mean macromolecular T_2_ as a function of the number of CEST offsets for the splenium, genu, frontal white matter (FWM), internal capsule (IC), caudate, frontal grey matter (FGM), and thalamus, with the qMT‐only fits as a comparison. Fits which are significantly different to the qMT‐only data are in bold (*P* < 10^−3^)
**FIGURE S1** Co‐registration process for the CEST data (every 2nd offset shown for clarity). The CEST data were first split into three groups of volumes using ±1 ppm as the demarcation point. The volumes greater than |1 ppm| were registered to the S_0_ image. Next, the volumes within ±1 ppm were registered to the co‐registered S(−1 ppm) image. The three volumes were then recombined for further processing
**FIGURE S2** B_1_ transmit map for an example volunteer. There are significant inhomogeneities in the anterior and posterior portions of the brain, which would significantly affect the CEST estimation maps if not corrected
**FIGURE S3** Correlation plots for each five pool (water, amide, hydroxyl, NOE, semisolid) full‐model fit as a function of both BSA concentration (A,C,E,G) and agarose concentration (B,D,F,H) for the amide (A,B), NOE (C,D), hydroxyl (E,F) and semisolid (E,F) pools. Dotted lines display the trendlines for the respective correlation. Introducing a hydroxyl pool results in strong correlations with BSA concentration for all pools when using the CEST+MT analysis, and strong correlations in all but the amide pool with agarose concentration. Similar trends can be seen in the qMT‐Fix analysis. Additionally, the NOE+MT analysis produces exactly the same correlations as the qMT‐Fix analysis
