# Supplementary material for: Does the magnetization transfer effect bias chemical exchange saturation transfer effects? Quantifying chemical exchange saturation transfer in the presence of magnetization transfer
Source: Magn Reson Med. 2020 Feb 18;84(3):1359–75. doi: 10.1002/mrm.28212 (PMC7317383; doi:10.1002/mrm.28212)
Supplement: Supplementary file 1 — TABLE S1 Pool Parameters used in simulations. T1 values were taken from the average of the T1 in the genu of a single subject. All other parameters were taken from van Zijl et al (1) TABLE S2 Mean free‐to‐macromolecular exchange rate as a function of the number of CEST offsets for the splenium, genu, frontal white matter (FWM), internal capsule (IC), caudate, frontal grey matter (FGM), and thalamus, with the qMT‐only fits as a comparison. Fits which are significantly different to the qMT‐only data are in bold (P < 10−3) TABLE S3 Mean macromolecular T2 as a function of the number of CEST offsets for the splenium, genu, frontal white matter (FWM), internal capsule (IC), caudate, frontal grey matter (FGM), and thalamus, with the qMT‐only fits as a comparison. Fits which are significantly different to the qMT‐only data are in bold (P < 10−3) FIGURE S1 Co‐registration process for the CEST data (every 2nd offset shown for clarity). The CEST data were first split into three groups of volumes using ±1 ppm as the demarcation point. The volumes greater than |1 ppm| were registered to the S0 image. Next, the volumes within ±1 ppm were registered to the co‐registered S(−1 ppm) image. The three volumes were then recombined for further processing FIGURE S2 B1 transmit map for an example volunteer. There are significant inhomogeneities in the anterior and posterior portions of the brain, which would significantly affect the CEST estimation maps if not corrected FIGURE S3 Correlation plots for each five pool (water, amide, hydroxyl, NOE, semisolid) full‐model fit as a function of both BSA concentration (A,C,E,G) and agarose concentration (B,D,F,H) for the amide (A,B), NOE (C,D), hydroxyl (E,F) and semisolid (E,F) pools. Dotted lines display the trendlines for the respective correlation. Introducing a hydroxyl pool results in strong correlations with BSA concentration for all pools when using the CEST+MT analysis, and strong correlations in all but the amide pool with agarose concen [file MRM-84-1359-s001.docx]

**Supporting Information Table S1:** Pool Parameters used in simulations. T_1_ values were taken from the average of the T_1_ in the genu of a single subject. All other parameters were taken from van Zijl et al (1).

|  | $\boldsymbol{M}_{\boldsymbol{0,r}}^{\boldsymbol{i}}$**(%)** | $\boldsymbol{k}_{\boldsymbol{iw}}$**(Hz)** | **T_1_ (ms)** | **T_2_ (ms)** | $\boldsymbol{\Delta}\boldsymbol{\omega}$ **(ppm)** |
| --- | --- | --- | --- | --- | --- |
| **Water** | 100 | ­ | 1050 | 80 | 0 |
| **Amide** | 0.5 | 30 | 1050 | 100 | 3.5 |
| **Creatine** | 0.5 | 1100 | 1050 | 170 | 2 |
| **NOE 1** | 0.5 | 16 | 1050 | 5 | -1.75 |
| **NOE 2** | 0.5 | 16 | 1050 | 5 | -2.75 |
| **NOE 3** | 0.5 | 16 | 1050 | 5 | -3.75 |
| **Semisolid** | 20 | 20 | 1050 | 10e-3 | -2.41 |

**Supporting Information Table S2:** Mean free-to-macromolecular exchange rate as a function of the number of CEST offsets for the splenium, genu, frontal white matter (FWM), internal capsule (IC), caudate, frontal grey matter (FGM), and thalamus, with the qMT-only fits as a comparison. Fits which are significantly different to the qMT-only data are in bold (p < 10^-3^).

|  | Number of CEST Offsets in Analysis | | | | | qMT-Only Data |
| --- | --- | --- | --- | --- | --- | --- |
|  | 41 | 31 | 21 | 11 | 1 |  |
| Splenium | **4.97±0.56** | **5.51±0.60** | **4.70±0.53** | **5.80±0.60** | 14.46±1.17 | 13.25±1.38 |
| Genu | **5.84±0.78** | **6.77±0.95** | **5.11±0.77** | **6.59±0.99** | 15.14±1.81 | 16.09±2.30 |
| FWM | **6.39±0.78** | **7.56±0.91** | **4.91±0.64** | **6.86±0.99** | 16.80±1.03 | 17.89±1.57 |
| IC | **4.76±0.63** | **5.32±0.68** | **4.71±0.64** | **5.93±0.74** | 15.78±1.04 | 16.34±2.18 |
| Caudate | **3.41±0.64** | **3.81±0.70** | **3.49±0.51** | **4.60±0.70** | 12.70±1.48 | 16.24±2.57 |
| FGM | **3.72±0.64** | **4.36±0.71** | **3.66±0.85** | **4.65±1.20** | **12.75±0.69** | 17.64±1.95 |
| Thalamus | **3.82±0.66** | **4.23±0.73** | **4.01±0.55** | **5.25±0.76** | **13.85±0.83** | 17.67±1.71 |

**Supporting Information Table S3:** Mean macromolecular T_2_ as a function of the number of CEST offsets for the splenium, genu, frontal white matter (FWM), internal capsule (IC), caudate, frontal grey matter (FGM), and thalamus, with the qMT-only fits as a comparison. Fits which are significantly different to the qMT-only data are in bold (p < 10^-3^).

|  | Number of CEST Offsets in Analysis | | | | | qMT-Only Data |
| --- | --- | --- | --- | --- | --- | --- |
|  | 41 | 31 | 21 | 11 | 1 |  |
| Splenium | **13.28±0.55** | **12.89±0.44** | **13.25±0.68** | **12.53±0.51** | 10.28±0.16 | 10.70±0.40 |
| Genu | **13.41±0.28** | **12.93±0.22** | **13.50±0.37** | **12.78±0.23** | 10.53±0.27 | 10.74±0.29 |
| FWM | **13.30±0.16** | **12.80±0.15** | **13.98±0.26** | **12.97±0.34** | 10.61±0.19 | 11.02±0.18 |
| IC | **14.19±0.34** | **13.68±0.27** | **14.28±0.37** | **13.38±0.23** | 10.58±0.07 | 11.41±0.19 |
| Caudate | **13.08±0.65** | **12.69±0.59** | **13.05±0.54** | **12.23±0.54** | 10.26±0.23 | 10.46±0.40 |
| FGM | **12.75±0.32** | **12.33±0.28** | **12.98±0.42** | **12.40±0.53** | 10.32±0.15 | 10.68±0.25 |
| Thalamus | **13.54±0.31** | **13.13±0.26** | **13.54±0.34** | **12.64±0.29** | 10.33±0.08 | 10.77±0.22 |


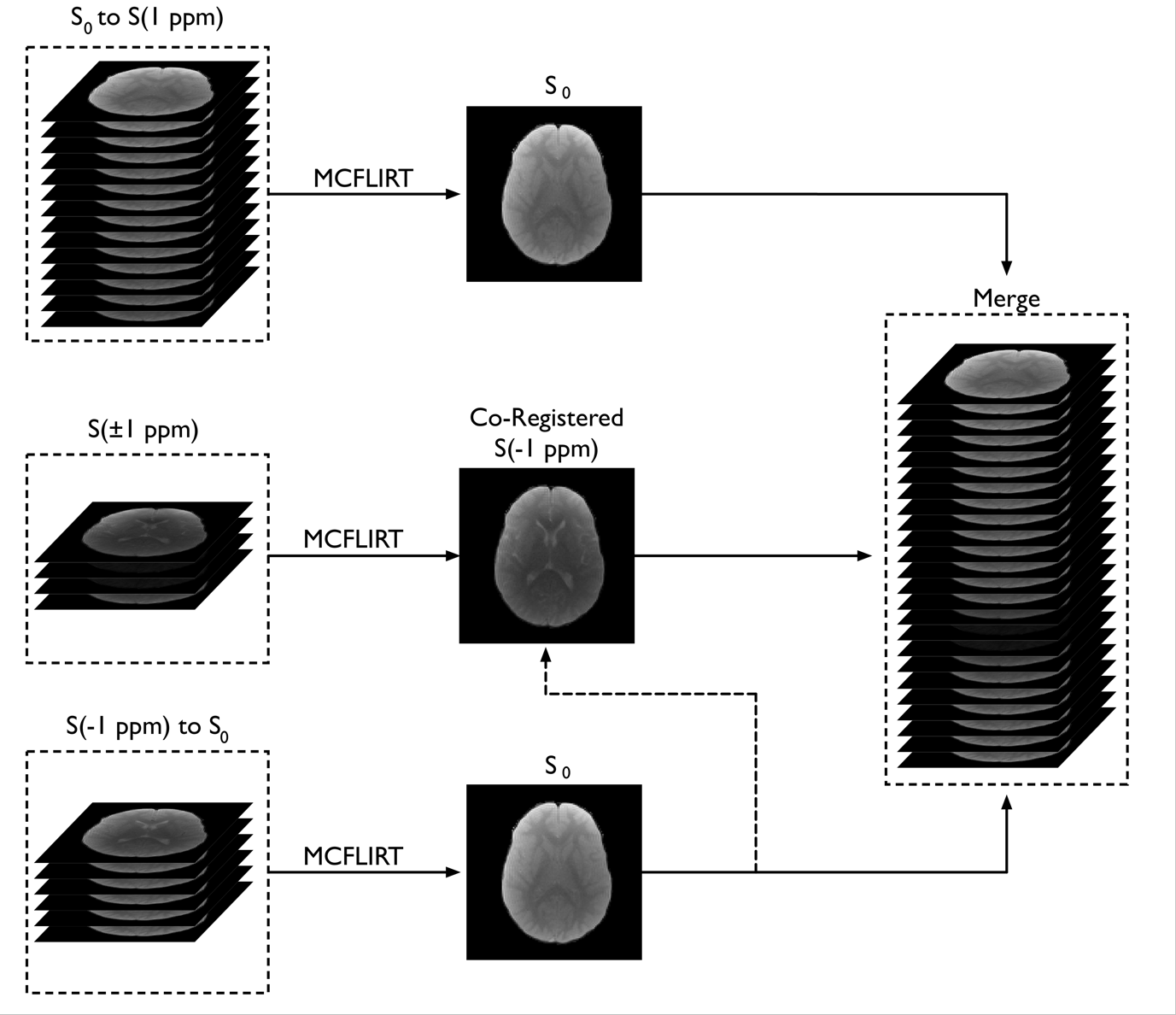


**Supporting Information Figure S1:** Co-registration process for the CEST data (every 2^nd^ offset shown for clarity). The CEST data was first split into three groups of volumes using ±1 ppm as the demarcation point. The volumes greater than |1 ppm| were registered to the S_0_ image. Next, the volumes within ±1 ppm were registered to the co-registered S(-1 ppm) image. The three volumes were then recombined for further processing.


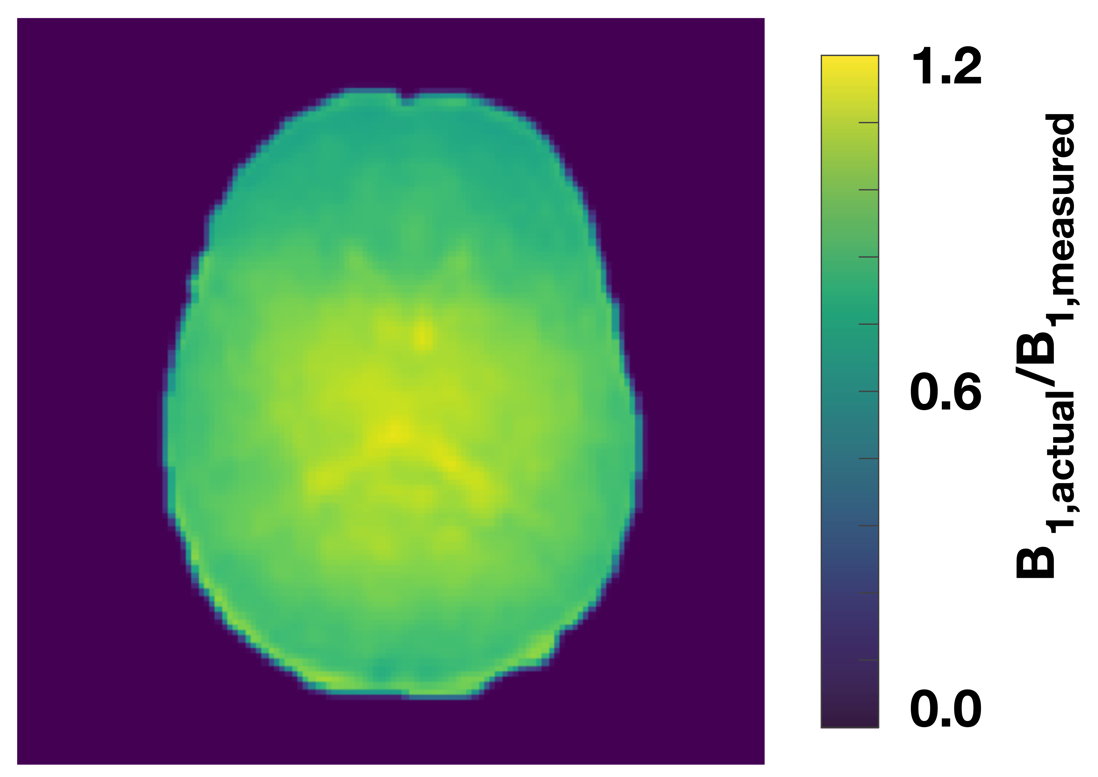


**Supporting Information Figure S2:** B_1_ transmit map for an example volunteer. There are significant inhomogeneities in the anterior and posterior portions of the brain, which would significantly affect the CEST estimation maps if not corrected.


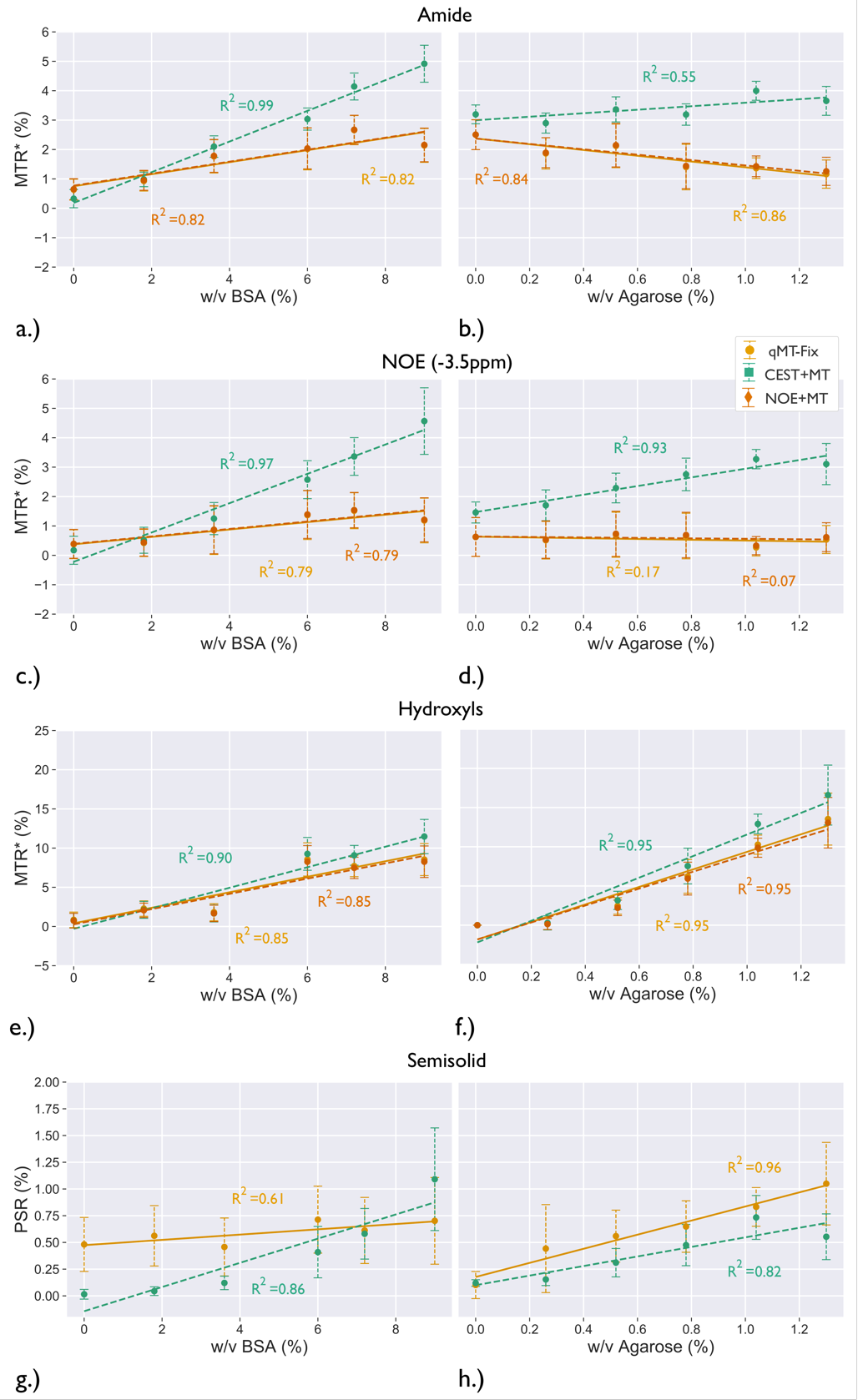


**Supporting Information Figure S3:** Correlation plots for each five pool (water, amide, hydroxyl, NOE, semisolid) full-model fit as a function of both BSA concentration (a, c, e, g) and agarose concentration (b, d, f, h) for the amide (a, b), NOE (c, d), hydroxyl (e, f) and semisolid (e, f) pools. Dotted lines display the trendlines for the respective correlation. Introducing a hydroxyl pool results in strong correlations with BSA concentration for all pools when using the CEST+MT analysis, and strong correlations in all but the amide pool with agarose concentration. Similar trends can be seen in the qMT-Fix analysis. Additionally, the NOE+MT analysis produces exactly the same correlations as the qMT-Fix analysis.

**References:**

1. van Zijl PCM, Lam WW, Xu J, Knutsson L, Stanisz GJ. Magnetization Transfer Contrast and Chemical Exchange Saturation Transfer MRI. Features and analysis of the field-dependent saturation spectrum. Neuroimage 2018;168:222–241 doi: 10.1016/j.neuroimage.2017.04.045.
